# Letter to the editor, “Effects of vitamin D deficiency on blood lipids and bone metabolism: a large cross-sectional study”

**DOI:** 10.1186/s13018-023-03773-x

**Published:** 2023-04-18

**Authors:** Changhua Liu, Laqing Chen

**Affiliations:** 1 Department of Orthopedics, Xiaoshan Affiliated Hospital of Wenzhou Medical University, Zhejiang, 311200 China; 2 Department of Respiration, Affiliated Xiaoshan Hospital, Hangzhou Normal University, Zhejiang, 311200 China

Dear Editor,

We have read with interest the article by Gu et al. [1] "Effects of vitamin D deficiency on blood lipids and bone metabolism: a large cross-sectional study". The authors conducted a large cross-sectional study using NHANES database and found that the increase in serum high-density lipoprotein is related to decreased bone mineral density of spine only in women aged 40–59 years with vitamin D insufficiency or deficiency. There is one question we would like to communite.

The authors analyzed data of NHANES 2007–2010. However, we found that data on 25(OH)D from these two surveys cannot be downloaded from the website (https://wwwn.cdc.gov/Nchs/Nhanes/Search/DataPage.aspx?Component=Laboratory&CycleBeginYear=2009). Accordingly, we would like to know how the authors got these data.

We appreciate the authors for their work on this valuable and instructive topic and hope that there will be more research in this area.

## Data Availability

Not applicable.
